# Naringin’s Prooxidant Effect on Tumor Cells: Copper’s Role and Therapeutic Implications

**DOI:** 10.3390/ph15111431

**Published:** 2022-11-19

**Authors:** Mohd Farhan

**Affiliations:** Department of Basic Sciences, Preparatory Year Deanship, King Faisal University, Al-Ahsa 31982, Saudi Arabia; mfarhan@kfu.edu.sa

**Keywords:** naringin, tumor microenvironment, copper, DNA damage, cell death

## Abstract

Plant-derived polyphenolic chemicals are important components of human nutrition and have been found to have chemotherapeutic effects against a variety of cancers. Several studies in animal models have proven polyphenols’ potential to promote apoptosis and tumor regression. However, the method by which polyphenols show their anticancer effects on malignant cells is not well understood. It is generally known that cellular copper rises within malignant cells and in the serum of cancer patients. In this communication, investigations reveal that naringin (a polyphenol found in citrus fruits) can strongly suppress cell proliferation and trigger apoptosis in various cancer cell lines in the presence of copper ions. The cuprous chelator neocuproine, which confirms copper-mediated DNA damage, prevents such cell death to a large extent. The studies further show that the cellular copper transporters CTR1 and ATP7A have a role in the survival dynamics of malignant cells after naringin exposure. The findings emphasize the crucial function of copper dynamics and mobilization in cancer cells and pave the path for a better understanding of polyphenols as nutraceutical supplements for cancer prevention and treatment.

## 1. Introduction

Cancer is a complex disease in which abnormal cells multiply as a result of normal cell proliferation and cell cycle processes being disrupted, resulting in tumors that expand and invade other body areas [1,2]. There are several cancer treatment options; however, some may be ineffective due to increased resistance to standard anticancer drugs and unwanted side effects [3]. Incorporating additional fruits and berries into the human diet, particularly citrus fruits, may benefit cancer prevention and progression [4,5,6]. Fruit-derived anticancer and therapeutic compounds, such as flavonoids and their derivatives, have shown a significant ability to inhibit tumor and cancer cell development [7]. Flavonoids have been shown in multiple studies to have potent anticancer activities by acting as antioxidants, changing ROS-scavenging enzyme activity, increasing apoptosis, autophagy, and cell cycle arrest, and decreasing inflammation, proliferative processes, and metastatic formation [3,8,9,10,11,12]. Naringin, a flavanone glycoside produced from naringenin, is found in several fruits of the Citrus genus, particularly grapefruit [13]. Naringin is thought to have a variety of pharmacological effects, including antioxidant, anti-inflammatory, anti-apoptotic, anti-tumor, and anti-viral characteristics [14,15,16,17]. Few papers exist that provide an overview of naringin in cancer without a focus on its anticancer qualities, and none have explored naringin separately in cancer prevention and treatment [17,18,19]. The mechanisms behind naringin’s anticancer activities are not fully known and are the focus of extensive research.

Copper is a metal ion found in chromatin that is tightly connected to DNA bases, specifically guanine [20]. It is one of the most redox-active metal ions found in living cells. Elevated copper levels have been observed in a variety of malignancies [21] in both humans and laboratory animals. Copper levels have been discovered to be raised in both the serum and tissue of malignant human tumors, which is a fascinating discovery [21]. The increase in copper in the tumor is not caused by the type of tissue but rather by a metabolic feature of the tumor itself [22].

The majority of plant-derived polyphenols have both antioxidant and prooxidant activities [23]. The prooxidant activity of plant-derived polyphenols, which mediates their selective anticancer action, is thought to be the result of a selective elevation in copper levels in malignant cells compared to non-cancerous controls. Plant-derived polyphenolics undergo a Fenton-like reaction with cellular copper in the presence of DNA, resulting in the production of reactive oxygen species (ROS) and apoptosis-like cell death [23].

**Figure 1:**
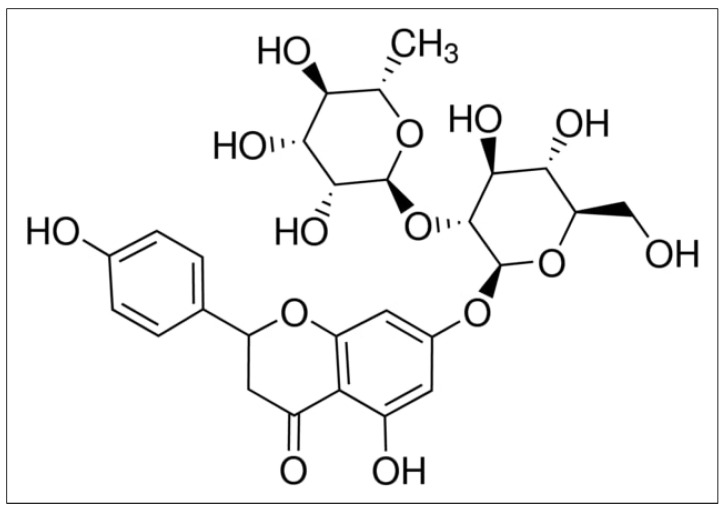
Chemical structure of naringin.

The powerful oxidative, damage-inducing ability of naringin is demonstrated in this study. It has been demonstrated that naringin’s abilities in malignant cells are reliant on the cellular bioavailability of copper and its redox recycling. Figure 1 depicts the chemical structure of naringin.

## 2. Results

### 2.1. Copper Chelation Inhibits Naringin-Induced Growth Inhibition

**Figure 2:**
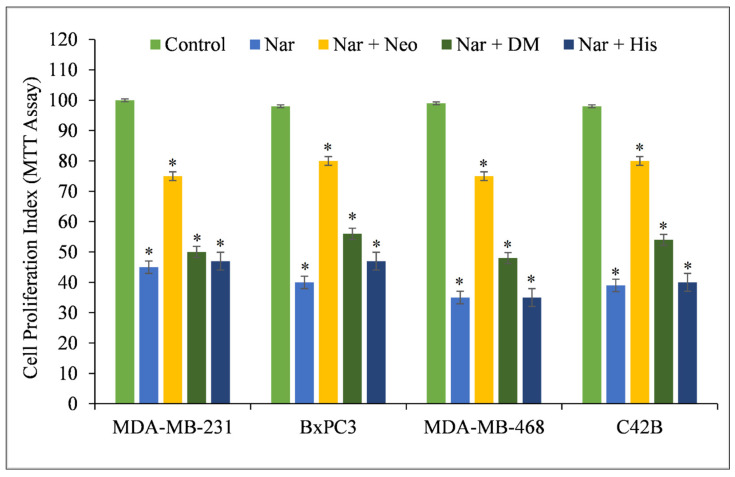
Effects of certain metal chelators on the antiproliferative action of naringin in four distinct cancer cell lines. As illustrated in the picture, cancer cells were treated with 100 µM naringin alone or in the presence of the copper chelator neocuproine (Neo), the iron chelator desferrioxamine mesylate (DM), or the zinc chelator histidine (His). The metal chelator concentration utilized was 50 µM. After 72 h of treatment, the MTT assay specified in the Materials and Methods section was conducted. Values reported are ±S.E.M. of three independent experiments. * *p*-value < 0.05 when compared to respective control.

Several studies show that naringin suppresses cell proliferation, migration, and invasion and enhances apoptosis in a variety of cancer cells, including those of bladder, hepatocellular, breast, colorectal, and gastric malignancies [24]. Copper appears to play a critical role in the cytotoxicity of naringin [25,26] based on the existing research. To confirm the critical role of intracellular copper in the cytotoxic action of naringin, multiple cancer cell lines were treated with specific metal chelators, and it was discovered that only the copper chelator, Neo, was able to protect MDA-MB-231, BxPC-3, MDA-MB-468, and C42B cells against the growth-inhibiting action of naringin (Figure 2). DM and His (iron and zinc chelators, respectively) had a protective effect against naringin-induced growth suppression. However, this was still less than Neo’s level of inhibition.

### 2.2. Copper Chelation Inhibits Naringin-Induced Apoptosis

**Figure 3:**
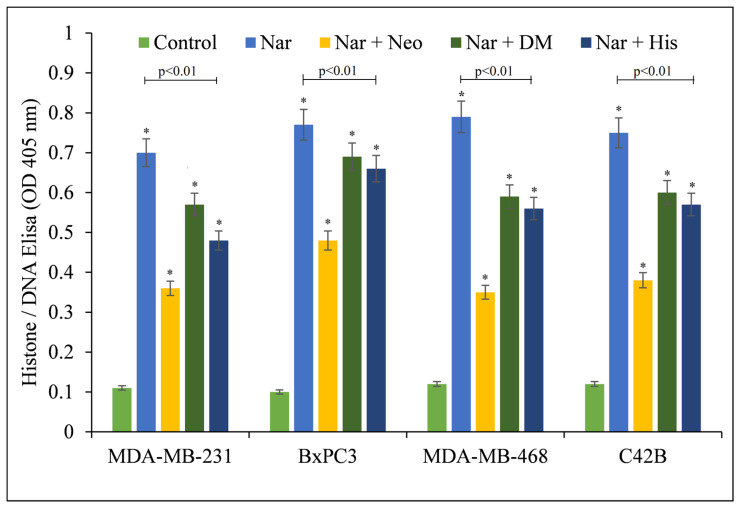
Effects of various metal chelators on naringin-induced apoptosis in four distinct cancer cell lines. MDA-MB-231, BxPC3, MDA-MB-468, and C42B cancer cells were treated with 100 µM naringin either alone or in the presence of the copper chelator neocuproine (Neo), the iron chelator desferrioxamine mesylate (DM), or the zinc chelator histidine (His), as shown in the figure. The metal chelator concentration utilized was 50 µM. As mentioned in the Materials and Methods section, ELISA was conducted 72 h following treatment. Values reported are ±S.E.M. of three independent experiments. * *p*-value < 0.05 when compared to respective control.

Additionally, the effect of various metal chelators on naringin-induced apoptosis was examined (Figure 3). The copper chelator neocuproine gave substantial protection. Iron and zinc chelators also showed some protective effects. However, this was still less than the protection with neocuproine, corroborating the notion that the anticancer mechanism of naringin includes copper mobilization.

### 2.3. Naringin Inhibits the Expression of Copper Transporters CTR1 and ATP7A

**Figure 4:**
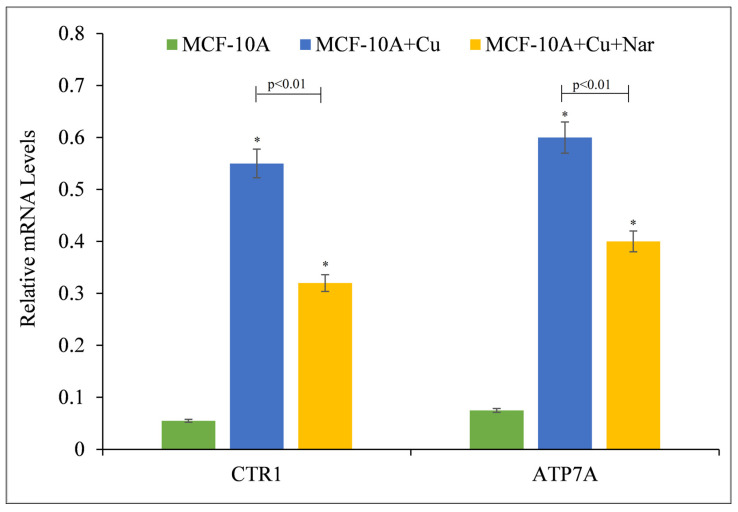
The effect of naringin on the elevated mRNA transcript levels of copper transporters CTR1 and ATP7A in MCF-10A-Cu cells compared to MCF-10A cells. Total RNA was extracted utilizing TRIzol reagent (Invitrogen, Carlsbad, CA, USA) per manufacturer’s instructions. CTR1 and ATP7A mRNA expression was quantified using real-time PCR, as stated in the Materials and Methods section. To determine the effect of naringin on mRNA expression, only MCF-10A-Cu (normal MCF-10A cells grown in a medium containing 25 µM CuCl_2_) was treated with 100 µM naringin. Values reported are ±S.E.M. of three independent experiments. * *p*-value < 0.05 when compared to untreated control.

It was revealed that naringin-induced growth inhibition and death in malignant cells is a result of the compound’s interaction with intracellular copper (Figure 2 and Figure 3). Due to the fact that malignant cells have a higher expression of copper transporter CTR1 and ATP7A [27], it was determined if copper supplementation led to an increase in copper transporter expression in non-malignant epithelial cells. Copper supplementation in the growth media of MFC-10A cells resulted in a significant upregulation of the copper transporters CTR1 and ATP7A, according to the findings (Figure 4). Further addition of naringin to the medium resulted in a reduction in the expression of both copper transporters, suggesting an influence of naringin on copper metabolism in cancer cells.

### 2.4. Targeted Silencing of CTR1 and ATP7A in MCF-10A Cells Grown in Copper Supplemented Medium Reduces Naringin-Induced Inhibition of Proliferation

**Figure 5:**
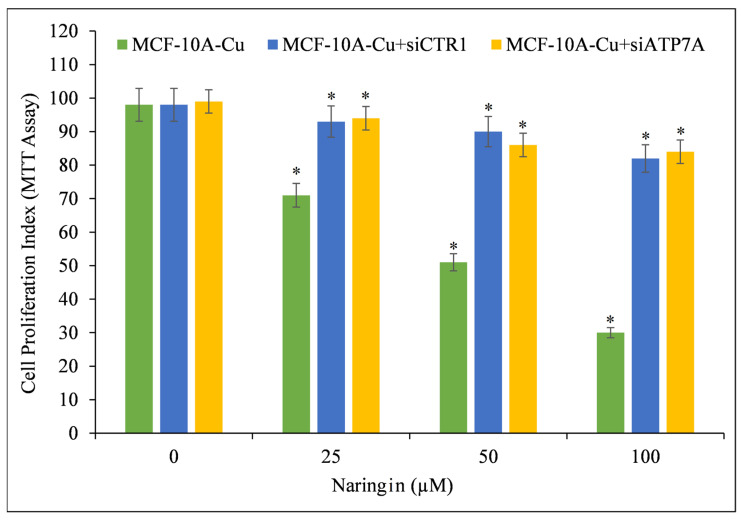
Cell proliferation of MCF-10A-Cu cells (normal MCF-10A cells cultured in a medium containing 25 µM CuCl_2_) was significantly reduced after treatment with naringin following CTR1 and ATP7A knock-down. MCF-10A-Cu cells were first treated with naringin or with specific si-RNA against CTR1 (siCTR1) and ATP7A (siATP7A) for 48 h and then with indicated concentrations of naringin for 24 h. Values reported are ±S.E.M. of three independent experiments. * *p*-value < 0.05 when compared to respective control.

Using targeted siRNA, copper transporter CTR1 and ATP7A were silenced to confirm copper’s crucial role in naringin-induced growth suppression (Figure 5). CTR1 and ATP7A mediate copper uptake in cells, and as established previously (Figure 4), their expression makes MCF-10A cells more susceptible to naringin-induced growth suppression. Copper-rich medium-grown MCF-10A cells were found to be less sensitive to naringin when the copper transporters CTR1 and ATP7A were silenced. This finding demonstrates conclusively that naringin interacts with cellular copper and that cellular copper is essential for naringin’s growth-inhibiting effect on cancer cells.

This firmly establishes that naringin interacts with cellular copper and that cellular copper is necessary for naringin’s growth-inhibiting impact on cancer cells.

## 3. Discussion

The prooxidant effect of plant-derived polyphenols, demonstrated by their interaction with intracellular copper and subsequent redox signaling [28,29,30], is one of the mechanisms by which polyphenols generate their selective lethal action. The observation that “normal” breast epithelial MCF-10A cells are resistant to naringin’s cytotoxic action compared to tumorigenic breast MDA-MB-231 and MDA-MB-468 cells is intriguing since it demonstrates the cancer cell selectivity of naringin’s cytotoxic action. The observation that MCF-10A cells become more sensitive to naringin-induced cytotoxicity when grown in the presence of copper confirms the crucial role of cellular copper in naringin-mediated physiological processes that lead to cell death. Copper’s physiological role in cancer is not well understood. Despite this, data indicates the importance of high copper levels in tumor angiogenesis [31]. Experiments have proven that plant-derived polyphenols interact with intracellular copper and induce oxidative DNA damage [32,33,34]. The new study provides additional support for this view. It has been demonstrated that naringin can inhibit angiogenesis [35]. It is likely that the anti-angiogenesis actions of naringin include copper mobilization and the resulting prooxidant effect; however, additional research is required to confirm this notion.

Normal breast epithelial cells cultivated in the presence of copper were shown to upregulate the copper transporters CTR1 and ATP7A, which were examined in the present work. Moreover, naringin has the potential to suppress the expression of these transporters. Thus, copper transporter expression correlates with the acquired susceptibility of epithelial cells to naringin activity. This fact adds an additional level of regulation to the hypothesis, in which naringin not only interacts with copper and causes oxidative DNA damage but also inhibits copper transporters, thereby impeding the copper metabolism of the “transformed” cell(s), which appears to be essential for their survival.

In addition, the results were validated through an experiment utilizing the siRNA-mediated inhibition of the expression of representative copper transporters CTR1 and ATP7A. Such silencing of CTR1 and ATP7A rendered MCF-10A cultured with copper supplementation insensitive to naringin, demonstrating and verifying that copper is necessary for naringin-induced selective cell death.

## 4. Materials and Methods

### 4.1. Cell Lines and Reagents

Naringin (Nar), metal chelators (neocuproine (Neo), bathocuproine disulphonic acid (Batho), desferrioxamine mesylate (DM) and histidine (His)), and cupric chloride (purity > 99%) were purchased from Sigma Chemical Co. (St. Louis, MO, USA). Cancer lines MDA-MB-231, MDA-MB-468, BxPC3, and C42B, as well as immortalized non-transformed breast cell line MCF-10A, were acquired from ATCC (Manassas, VA, USA). MDA-MB-468 and C42B cells were maintained in RPMI, whereas MDA-MB-231 and BxPC3 cell lines were maintained in DMEM (Invitrogen, Carlsbad, CA, USA). Then, 10% fetal bovine serum (FBS), 100 units/mL penicillin, and 100 µg/mL streptomycin were added to the medium. At 37 degrees Celsius and 5% CO_2_ (humid atmosphere), all cells were cultured. Naringin (50 mM) stock solutions were stored at −80 °C in tiny aliquots. The stock solutions of several metal ion chelators, such as neocuproine, desferoxamine mesylate, and histidine, were always generated freshly right before studies at a 50 mM final concentration in PBS. The normal breast epithelial cell line MCF-10A was cultured in DMEM/F12 (Invitrogen, Carlsbad, CA, USA) along with 5% horse serum, 20 ng/mL EGF, 0.5 µg/mL hydrocortisone, 0.1 µg/mL cholera toxin, 10 µg/mL insulin, 100 units/mL penicillin, and 100 µg/mL streptomycin. MCF-10A cells that have grown for a month in regular culture medium with 25 µM CuCl_2_ added are known as MCF-10A-Cu cells. All other chemicals were commercial products of analytical grade.

### 4.2. Cell Growth Inhibition Studies Using the 3-(4,5-Dimethylthiazol-2-yl)-2,5 Diphenyltetra-zolium (MTT) Assay

Subsequently, 2 × 10^3^ cells were seeded in each well of 96-well microtiter plates. The regular growing medium was replaced with a fresh medium containing varying concentrations of diluted 50 mM naringin stock after overnight incubation. As described in each study, specific assays were treated with a metal chelator. After 3 days of incubation, 25 µL of MTT solution (5 mg/mL in PBS) was added to each well, and plates were incubated for an additional 2 h at 37 °C and 5% CO_2_.

The supernatant was removed after a 2-h incubation period. Using a gyrating shaker, metabolically viable cell-derived MTT formazan was dissolved in 100 µL of DMSO for 30 min. Using an Ultra Multifunctional Microplate Reader, 595 nm was calculated as the absorbance (TECAN, Durham, NC, USA). Eight replicate wells were utilized for each treatment, and the DMSO concentration never surpassed 0.1%. Each experiment was conducted thrice.

### 4.3. Apoptosis Detection Using the Histone/DNA ELISA

Using the Cell Death Detection ELISA Kit (Roche, Palo Alto, CA, USA), apoptosis in growth cells treated with naringin was identified. The cells were treated for 72 h with naringin or DMSO as a control. After treatment, DNA and cytoplasmic histone were extracted from cells and incubated on microtiter plate modules coated with anti-histone antibody. Peroxidase-conjugated anti-DNA antibody was utilized to detect immobilized histone/DNA, followed by color development using a peroxidase-specific ABTS substrate. Using an Ultra Multifunctional Microplate Peruser (TECAN, Durham, NC, USA) at 405 nm, the spectrophotometric absorbance of the samples was measured.

In addition, various metal ion chelators were utilized during the reactions. DM (50 µM) was employed to chelate Fe (II), His (50 µM) to chelate Zn (II), and Neo (50 µM) to chelate Cu (II) ions.

### 4.4. Real-Time Reverse Transcriptase PCR

TRIzol reagent (Invitrogen) was used to isolate total RNA in accordance with the manufacturer’s instructions. To quantify mRNA expression, real-time PCR was performed. Sequences of primers for CTR1 (forward: 5′-GCT GGA AGA AGG CAG TGG TA-3′; reverse: 5′-AAA GAG GAG CAA GAA GGG ATG-3′), ATP7A (forward: 5′-ACG AAT GAG CCG TTG GTA GTA-3′; reverse: 5′-CCT CCT TGT CTT GAA CTG GTG-3′) and GAPDH (glyceraldehyde-3-phosphate dehydrogenase) (forward: 5′-TGG GTG TGA ACC ATG AGA AGT-3′; reverse: 5′-TGA GTC CTT CCA CGA TAC CAA-3′) were the same as reported earlier [36,37], and the amount of RNA was normalized to GAPDH expression.

### 4.5. Small Interfering RNA (siRNA) Transfection

siRNA transfections were carried out as outlined before [37]. Santa Cruz Biotechnology, Inc. was reached out to for siRNA targeting CTR1 and ATP7A. As a control, garbled siRNA was utilized. Copper transporters, i.e., CTR1 and ATP7A, were silenced by siRNA (0.60 nmol/μL) 48 h prior to the experiment utilizing Lipofectamine RNA iMAX Transfection Reagent (Invitrogen) as per the manufacturer’s guidelines.

### 4.6. Statistical Analysis

The statistical analysis was conducted as outlined by Tice et al. [38] and is expressed as the standard error of the mean ± S.E.M. of three independent experiments. A Student’s *t*-test was used to examine statistically significant differences. ANOVA was used to conduct an analysis of variance. *p*-values ≤ 0.05 were considered statistically significant.

## 5. Conclusions

It is possible to draw the conclusion that the availability of intracellular copper and the presence of polyphenols (such as naringin) affect their ability to cause oxidative DNA damage in cancer cells. The critical significance of intracellular copper levels, made possible by copper transporters, in the anticancer effect of naringin in particular and the plant-derived polyphenols, in general, has been established by the presented results. This adds a new dimension to the design of future mechanism-based research aimed at targeting the tumor microenvironment for the desired efficacy of non-toxic anticancer agents.

## Data Availability

Not applicable.
